# Resistant Genes and Multidrug-Resistant Bacteria in Wastewater: A Study of Their Transfer to the Water Reservoir in the Czech Republic

**DOI:** 10.3390/life12020147

**Published:** 2022-01-20

**Authors:** Tereza Stachurová, Nikola Sýkorová, Jaroslav Semerád, Kateřina Malachová

**Affiliations:** 1 Department of Biology and Ecology, Faculty of Science, University of Ostrava, Chittussiho 10, CZ-710 00 Ostrava, Czech Republic; P19073@student.osu.cz (N.S.); katerina.malachova@osu.cz (K.M.); 2 Institute of Microbiology of the Czech Academy of Sciences, Vídeňská 1083, CZ-142 20 Prague, Czech Republic; jaroslav.semerad@biomed.cas.cz; 3 Institute for Environmental Studies, Faculty of Science, Charles University, Benátská 2, CZ-128 01 Prague, Czech Republic

**Keywords:** antibiotic resistance gene, beta-lactam resistance, tetracycline resistance, multidrug resistance, wastewater, wastewater treatment plant, water environment

## Abstract

Wastewater is considered the most serious source of the spread of antibiotic resistance in the environment. This work, therefore, focuses on the fate and spread of antibiotic resistance genes (ARGs) in wastewater and the monitoring of multidrug-resistant strains. ARGs were monitored in the nitrification and sedimentation tanks of the wastewater treatment plant (WWTP) and in the dam into which this WWTP flows, at various times. The highest relative abundance was found for the *bla*TEM > *tet*W > *bla*NDM-1 > *van*A resistance genes, respectively. An increased concentration of tetracycline (up to 96.00 ng/L) and ampicillin (up to 19.00 ng/L) was found in water samples compared to other antibiotics detected. The increased incidence of seven ARGs and four antibiotics was observed in the November and December sampling times. Isolated ampicillin-resistant strains showed a high degree of resistance to ampicillin (61.2% of the total isolates had a minimum inhibitory concentration (MIC) ≥ 20 mg/mL). In 87.8% of isolates, out of the total number, the occurrence of two or more ARGs was confirmed. These multidrug-resistant strains were most often identified as *Aeromonas* sp. This strain could represent a significant role in the spread of multidrug resistance through wastewater in the environment.

## 1. Introduction

Antibiotic resistance is now considered a serious public health problem as it prevents the effective treatment of bacterial infections and can cause high mortality [1]. High consumption of antibiotics, their improper disposal, and excretion of drugs by humans and animals are the main reasons for the penetration of antibiotics and their metabolites into wastewater [2]. The main collectors of municipal and hospital wastewater are wastewater treatment plants (WWTPs) [3]. Due to their specific properties and technological functioning, WWTPs are determined to be one of the most serious reservoirs and sources of resistance. These are places where there may be an accumulation of antibiotics, antibiotic-resistant bacteria (ARB), and antibiotic-resistant genes (ARGs), but also an intense spread of antibiotic resistance among bacterial species [4,5,6]. It is important to understand the differences in the presence of ARG and ARB in the WWTP treatment phases at different times. It is thought that strong seasonal differences in antibiotic use may lead to seasonal changes in the level of antibiotic resistance in wastewater [7,8]. In addition, WWTPs have been shown not to completely eliminate ARGs and are an important source of the expansion of ARGs to various environmental compartments [9,10,11,12]. For example, the *bla*TEM resistance gene, one of the most abundant ARGs, has been found in wastewater but also in purified wastewater [13].

The transmission of resistance to pathogenic species poses a strong threat to human health [2]. Particularly dangerous are multidrug-resistant bacteria, which can carry resistance genes to multiple types of antibiotics. The most frequently identified bacterial strains bearing multidrug-resistant properties include in particular *Escherichia coli*, *Klebsiella pneumoniae*, *Staphylococcus aureus*, *Pseudomonas* spp., and *Proteus* spp., which can thus cause serious infections [14,15]. From the available information, multidrug resistance is mainly associated with the hospital environment [16,17], but multidrug-resistant strains can also be found in wastewater, which can have serious consequences not only for human health but also for the negative development of common microflora in the environment [18,19]. It is well known that wastewater treatment technology reduces the number of bacteria in wastewater [20,21], but is not effective enough to remove ARB and ARGs [22]. Therefore, it is important to not only monitor the occurrence of multidrug-resistant strains and their ARGs content in wastewater but also their ecological impact on neighboring water reservoirs, which are affected by these WWTPs.

The aim of this study was to monitor the presence of seven resistance genes (*bla*TEM, *bla*NDM-1, *bla*KPC, *bla*OXA-48, *mec*A, *tet*W, *van*A) and four antibiotics, which are among the most widely used antibiotics in the Czech Republic (ampicillin, penicillin G, penicillin V, and tetracycline) [23,24] in wastewater at each phase of the treatment processes of WWTPs and to monitor the spread of these ARGs and antibiotics into the dam water, where the WWTP discharges the treated wastewater. These genes have been selected as frequently monitored resistance genes in wastewater [25]. The influence of different time samples on the presence of ARGs and ARB was evaluated. Moreover, isolated resistant bacteria from water samples were characterized by minimum inhibitory concentration (MIC) and minimum bactericidal concentration (MBC) values for the antibiotics ampicillin and tetracycline. The study provides new data on the occurrence and identification of multidrug-resistant bacteria and the detection of ARGs not only in the wastewater of the WWTP but also in the neighboring water dam, which is affected by the WWTP. These data may be important to emphasize the impact of ARGs and ARB on the health of the population in the vicinity of the water reservoir in the Moravian-Silesian Region of the Czech Republic since this reservoir is used for recreation of the inhabitants and is also used for fish farming and sport fishing.

## 2. Materials and Methods

### 2.1. Wastewater and Environmental Sampling

**Figure 1:**
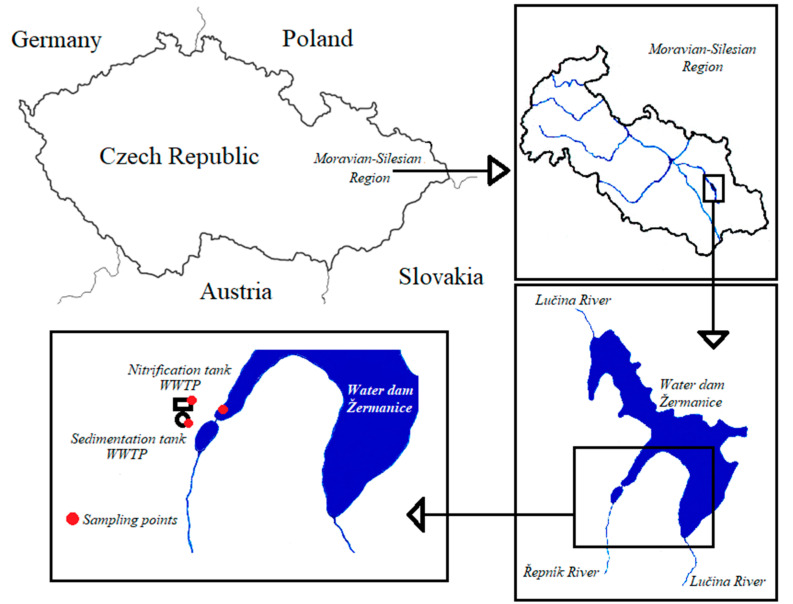
Overview of the study area and sampling points in the WWTP and water dam Žermanice.

Wastewater (WW) samples were obtained from the nitrification and sedimentation tanks of the WWTP (Moravian-Silesian Region, Czech Republic), comprising mechanical and biological treatments, and secondary sedimentation (Figure 1). The WWTP’s population equivalent (PE) is 1980 inhabitants. The biochemical oxygen demand (BOD) of the inflow and outflow is 198 and 115 mg/L, respectively; daily peak flow is 4.3 L/s. Inflowing water contains a mixture of municipal and agricultural WW, whereas the passed water discharges in the water dam Žermanice. Water dam Žermanice is an important source of water for the supply of companies in the Moravian-Silesian region, for the production of electricity, and it is widely used for recreation. Due to the extensive use of this water dam, it is necessary to monitor the dam to prevent negative effects on human health. Another water sample was taken from the water dam located in the vicinity of the WWTP effluent. Each sample was merged as five aliquots from different parts of the tank/dam. The sampling A, B, and C was carried out in December 2019, August 2020, and November 2020, respectively. The samples were taken on ambient light and the water temperature ranged from 12.1–19.3 °C, and 2 L of total sample volumes was transported in a refrigerated container at 4 °C. In the laboratory, the samples were immediately stored at 4 °C and processed no later than 24 h.

### 2.2. Determination of Counts of Non-Resistant and Resistant Bacteria

WWTP WW and dam-water samples were serially diluted. The original sample of the WW from the nitrification tank had a dilution factor of 1000, the WW from the sedimentation tank and the dam water was diluted by a dilution factor of 10. Two hundred microliters of diluted samples was loaded on tryptic soy agar (TSA, Sigma-Aldrich, St. Louis, MI, USA) and TSA containing antibiotic ampicillin (AMP, Sigma-Aldrich). According to previous research, the ampicillin concentration of 500 mg/L was introduced [20]. Plated samples were incubated at 30 °C for 24 h; the counts of formed bacterial colonies were used to determine the colony-forming units (CFU) per mL of the water sample. Isolated ampicillin-resistant bacteria were stored at −80 °C. The cultivations were performed in three independent batches.

### 2.3. Measurement of Growth Curves of Resistant Isolates

The growth curves and growth curve parameters were determined for all ampicillin-resistant isolates obtained. The bacterial suspension from each isolate was diluted to 0.1 for OD600 in tryptic soy broth (TSB, Sigma-Aldrich). The absorbance at OD600 was measured every 30 min, at 30 °C for 24 h (Bioscreen C MBR, Helsinki, Finland).

### 2.4. Determination of MIC and MBC Values

A total of 49 ampicillin-resistant bacterial isolates were characterized by MIC and MBC for antibiotics ampicillin and tetracycline (Fluka BioChemika, Buchs, Switzerland). MIC values were determined using the microdilution method described previously [26]. Briefly, a dilute inoculum in TSB (0.1 at OD600) was prepared from all resistant isolates. The bacterial inocula were applied to a 96-well plate. The inocula were exposed to the ampicillin concentration range of 0.625–20.0 mg/mL and the tetracycline concentration in the range of 0.25–16.0 µg/mL. The TSB medium was used as a negative control; a dilute isolate to 0.1 at OD600 without added antibiotic was used as a positive control. After 24 h of incubation at 30 °C, the absorbance at OD600 was measured. MIC was characterized as the lowest antibiotic concentration where the turbidity extinctions of bacterial suspension were observed. To evaluate MBC values, 10 μL of the non-turbid isolate suspension was loaded onto TSA agar medium and cultured for 24 h at 30 °C. MBC was defined as the lowest antibiotic concentration, which is able to kill all bacteria.

### 2.5. Antibiotic Determination

Water samples (nitrification and sedimentation tank of WWTP, water dam) were filtered using filters with 0.22 µm pore size (Pall Corporation, Mexico City, Mexico), preconcentratated by solid-phase extraction, and detected using ultra-performance liquid chromatography combined with triple quadrupole mass spectrometry (UHPLC-MS/MS) according to a method already described [27]. The detection limit for determining the level of antibiotics was 0.05 ng/L.

### 2.6. Isolation of Bacterial DNA from Water

WWTP WW and dam-water samples were filtered through membrane filters (20 mL of WW from the nitrification tank, 200 mL of WW from the sedimentation tank, and 200 mL of water from the dam). The volume of filtered samples from the sedimentation tank and the dam was increased due to an insufficient amount of DNA. The filters were preserved at −80 °C. To extract DNA from the filters, a suspension of 1 mol/L CaCO_3_ was added to the filters and incubated overnight at 4 °C. Subsequently, bacterial DNA was isolated according to a previously described method (method number 4) [28]. The purity and concentration of the isolated DNA were analyzed using the Nanophotometer P300 (IMPLEN, Munich, Germany). DNA samples were frozen at −20 °C.

### 2.7. Relative Abundance of ARGs in Water Samples

The ARGs (*bla*TEM, *bla*NDM-1, *bla*KPC, *bla*OXA-48, *mec*A, *tet*W, and *van*A) were analyzed from the bacterial DNA extracted from the WW and dam-water samples (see Section 2.6). Primers and qPCR conditions are listed in Table S1 [29,30,31,32,33,34]. The qPCR mixture was composed of 18 µL Xceed qPCR SG 2x Mix Lo-ROX (Institute of Applied Biotechnologies, Prague, Czech Republic) and 2 µL of extracted DNA. The amount of gene was converted to the total content of the 16S rRNA gene in the individual samples.

pMOS plasmids, containing fragments of quantified resistance genes and 16S rRNA, were serially diluted 10-fold. For each standard curve, the PCR amplification efficiency (E) was calculated from the following Equation (1):

(1) $$E=10^{\left(\frac{-1}{\mathrm{slope}}\right)}-1\times \left(100\%\;\mathrm{efficiency}=1\right)$$

### 2.8. Evaluation of Multidrug Resistance of Bacterial Isolates

Multidrug resistance was evaluated in all 49 ampicillin-resistant isolates. The genomic DNA was isolated from the bacterial isolates according to a previous method [28]. Resistance genes to beta-lactam (*bla*TEM, *mec*A), carbapenem (*bla*NDM-1, *bla*KPC, *bla*OXA-48), tetracycline (*tet*W), and vancomycin (*van*A) were detected. The primers used are listed in Table S1.

### 2.9. Identification of Resistant Bacteria

Ampicillin-resistant isolates were identified by 16S rRNA gene sequencing. Then, 16S rRNA fragments were amplified from the genomic DNA of bacterial isolates using universal bacterial primers 1378R and 984F (Table S1) [35]. The final PCR products were purified. Unidirectional sequencing was performed by The Center for DNA Sequencing (SeqMe s.r.o., Dobříš, Czech Republic). The sequences of bacterial isolates obtained were assigned the closest results using BLASTn from the NCBI database (www.ncbi.nlm.nih.gov/, accessible from 19 December 2021). Each sequence was stored in GeneBank NCBI with its individual accession number.

### 2.10. Statistical Analysis

All experimental data were obtained as the mean of triplicates ± standard deviation, unless otherwise stated. CFU/mL data were compared between the presence of non-resistant and resistant bacteria in water samples, between individual sampling sites, and different sampling times using Tukey’s post hoc test [36]. An ANOVA test was also performed, which was used to evaluate the differences between the amounts of quantified ARGs in water samples [10]. Significant associations between the presence of ARGs in multidrug-resistant strains, place, and time of sampling were tested by chi-square analysis [37]. The Growthcurver package was applied to compute the growth curves and parameters of the growth curves of bacterial isolates. *p* < 0.05 was determined to be statistically significant. The statistical tests used in this study were performed in the R program (R Core Team, 2016, version 3.4.0, The R Foundation for Statistical Computing, Vienna, Austria).

## 3. Results and Discussion

### 3.1. Antibiotic Determination

**Figure 2:**
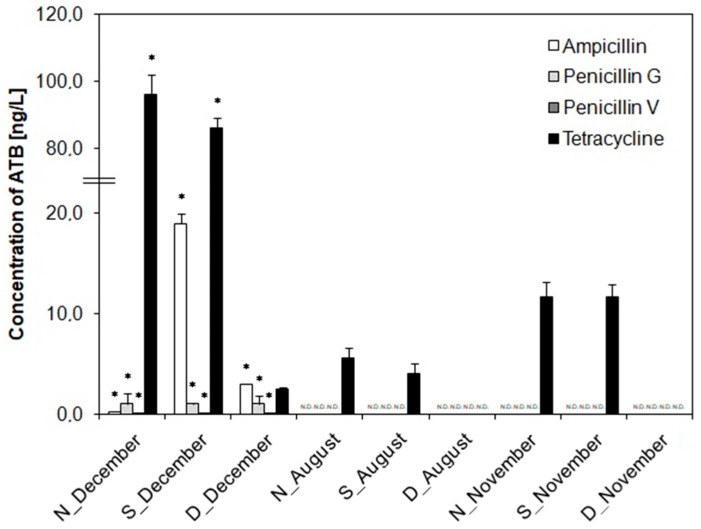
The concentrations of ampicillin, penicillin G, penicillin V, and tetracycline analyzed in the water samples at different sampling campaign using UHLPC-MS/MS. Detection limit = 0.05 ng/L. ATB, antibiotic; N, nitrification tank; S, sedimentation tank; D, dam; *, value at *p* < 0.05 indicates significant difference between different sampling times in the same sampling site; N.D., no antibiotics were detected.

To observe the effectiveness of the WWTP in removing antibiotics during treatment processes and monitoring the effect of antibiotics on the emergence of resistant strains, the levels of the antibiotics ampicillin, penicillin G, penicillin V, and tetracycline were analyzed in all water samples. Ampicillin, penicillin G, and penicillin V were detected only in sampling campaign A (Figure 2). In other sampling campaigns, the values of these antibiotics were below the detection limit. Penicillin G and V were detected at the highest concentrations of 1.0 and 0.1 ng/L, respectively (Figure 2). For ampicillin, concentrations of 0.25 ng/L were detected in the nitrification tank WWTP, 19.0 ng/L in the sedimentation tank WWTP, and 3.0 ng/L in the dam water. Thus, it can be assumed that ampicillin plays a relevant role in the accumulation of antibiotic resistance in the sedimentation tank. Kulkarni et al. (2017) also found similar results regarding the accumulation of ampicillin in sedimentation tanks [38]. However, the highest concentrations were found for tetracycline in all sampling campaigns: up to 96 ng/L in the nitrification tank WWTP in sampling campaign A, up to 5.6 ng/L in the nitrification tank WWTP in sampling campaign B, and 11.7 ng/L in the nitrification and sedimentation tank of the WWTP in sampling campaign C (Figure 2). High tetracycline concentrations in wastewater treatment plants have generally been measured in the range of 96–1300 ng/L [39]. Our study showed a decrease in tetracycline concentrations in all dam waters, which can be considered as a high effect of the WWTP treatment process. In terms of sampling time differences, a significant difference was found in sampling campaign A (December), where all antibiotics were detected, compared to other sampling times (*p* < 0.05).

It cannot be ruled out that the presence and accumulation of trace antibiotic concentrations in different WWTP tanks may cause the production of antibiotic resistance genes in bacteria [40].

### 3.2. Quantification of ARGs in Water

To further study the development of antibiotic resistance, we quantified the genes mediating antibiotic resistance by qPCR in all water samples. Resistance genes were monitored *bla*TEM, *bla*NDM-1, *bla*KPC, *bla*OXA-48, *mec*A, *tet*W, and *van*A. The R^2^ values (0.993–0.998) and efficiencies (92.66% to 99.26%) calculated from the standard curves characterizing the qPCR assays are shown in Table S2.

**Figure 3:**
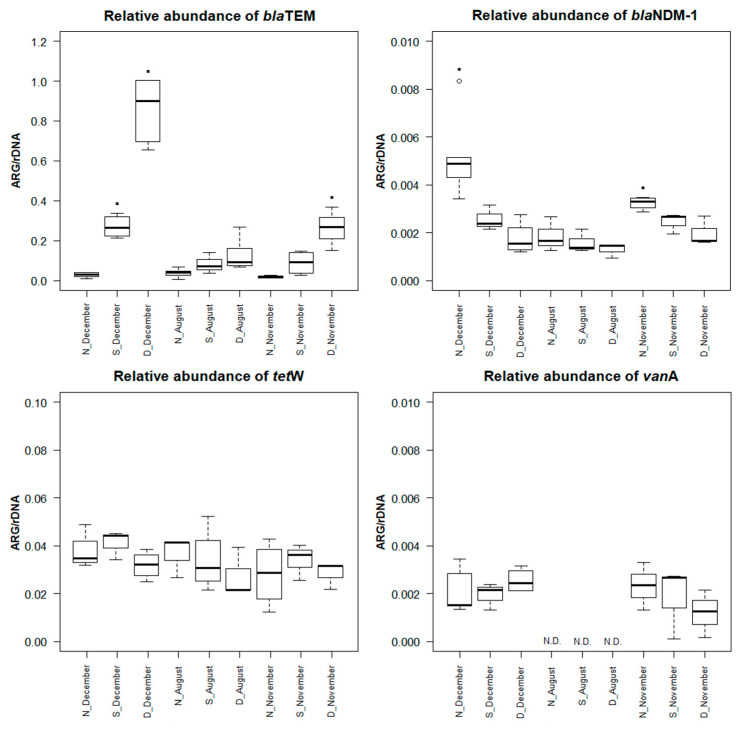
The relative abundance of antibiotic resistance genes (*bla*TEM, *bla*NDM-1, *tet*W, and *van*A) in the water samples at different sampling campaign. N, nitrification tank; S, sedimentation tank; D, dam; *, significant value at *p* < 0.05; N.D., no antibiotics were detected.

Of all the genes tested, *bla*TEM, *bla*NDM-1, *tet*W, and *van*A were detected. The highest relative abundance of all genes was confirmed in *bla*TEM (Figure 3). This is not surprising, as *bla*TEM has previously been categorized into so-called “frequently abundant” genes in wastewater, as it represents 99.9% of the total number of detected ARGs [41]. It has also been proven several times in Czech wastewater [20,27]. In our study, *bla*TEM always showed the highest relative abundance in dam water in all sampling campaigns, mostly in sampling campaign A (*p* < 0.05) (Figure 3). This may be related to the high level of the antibiotic ampicillin in the same sampling campaign (Figure 2). Despite the probable reduction in the absolute amount of bacteria after the WWTP treatment processes, this gene most often occurred in the treated water. Rafraf et al. (2016) also reported an increase in ARGs after wastewater treatment [42]. A possible explanation for the observed increases in *bla*TEM in the dam water may be the accumulation of beta-lactam-resistant bacteria in the WWTP tanks and the subsequent release of the *bla*TEM gene into the environment.

A completely opposite trend was shown for the *bla*NDM-1 gene (Figure 3). The largest relative abundance was found in the nitrification tank in all sampling campaigns. Again, the highest values of relative abundance were quantified in sampling campaign A (*p* < 0.05). Subsequently, the relative abundance of this gene decreased both in the sedimentation reservoir and in the dam. This indicates sufficient WWTP processes that have been sufficient in partially blocking the spread of this gene into the environment. The *bla*NDM-1 gene provides resistance to a broad spectrum of carbapenems [43]. It is considered a rare ARG (only 0.0001% of the total ARGs) [41]. Recently, however, it has become increasingly common in wastewater [20,44,45], indicating a high degree of risk of carbapenem resistance.

The *tet*W gene was quantified with the second-highest relative abundance of all genes detected in our study (Figure 3). The relatively frequent occurrence in wastewater of this gene is well known [27,37]. In all sampling campaigns, *tet*W had an approximate relative abundance, i.e., that the sampling time difference had no effect on the occurrence of this gene (*p* > 0.05). However, in sampling campaigns A and C, there was a slight increase in the relative abundance of the *tet*W gene in the sedimentation tanks of the WWTP (Figure 3). Increasing the amount of the *tet* gene from the anaerobic tank to the secondary settling tank of the WWTP has previously been found by Liu et al. (2019) [46].

The *van*A gene was quantified only in sampling campaigns A and C (Figure 3). In both campaigns, its trend is completely different. In sampling campaign A, its relative abundance increases, while in sampling campaign C, the relative abundance of the *van*A gene decreases (Figure 3). However, it is important that this gene enters the environment despite the WWTP processes. The presence of bacteria with the *van*A gene in treated wastewater from WWTPs was confirmed in an earlier study from the Czech Republic [47]. *van*A, like *bla*NDM-1, is a rare gene in effluent [41]. *van*A is responsible for the high level of resistance to the antibiotic vancomycin and is one of the most commonly acquired mechanisms of resistance in Europe [48]. However, the recurrence of vancomycin-resistant bacteria and the *van*A gene in treated wastewater may increase the risk of incorporation into the environment.

Sampling time differences affected the occurrence of *bla*TEM, *bla*NDM-1, and *van*A genes in wastewater and subsequently in the recipient (*p* < 0.05). Higher relative abundances were observed mainly in sampling campaign A (December) and subsequently in sampling campaign C (November). Caucci et al. (2016) also suggested the same seasonal fluctuations in ARGs [49]. This phenomenon can be explained by the different seasonal dynamics of prescribing individual antibiotics [7].

### 3.3. Characteristics of Ampicillin-Resistant Bacteria from Water Samples

The presence of non-resistant and ampicillin-resistant bacterial strains in water samples was monitored by determining CFU/mL (Table 1). A significant statistical difference between resistant and non-resistant bacteria was confirmed for all samples (*p* < 0.05) except: nitrification tank WWTP (sampling campaign A) and nitrification and sedimentation tank WWTP (sampling campaign C). This agrees with our results described above, where we found a higher relative abundance of detected ARGs in water samples in sampling campaigns A and C (Figure 3). Increased bacterial growth was observed in the nitrification tank of WWTP compared to other sampling sites (*p* < 0.05) (Table 1). It was found that the occurrence of bacteria was not affected by sampling time differences (*p* > 0.05). This finding is consistent with previous studies [20,21].

Bacterial strains growing on TSA containing 500 mg/L ampicillin were isolated. A total of 49 ampicillin-resistant bacteria were isolated. These isolates were marked according to the sampling site (nitrification tank WWTP—N, sedimentation tank WWTP—S, dam—D) and according to sampling time (A—December, B—August, C—November). Growth curves and growth curves parameters (growth rate, lag phase, doubling time) of ampicillin-resistant isolates are summarized in Supplementary Materials (Figure S1, Table S3).

**Figure 4:**
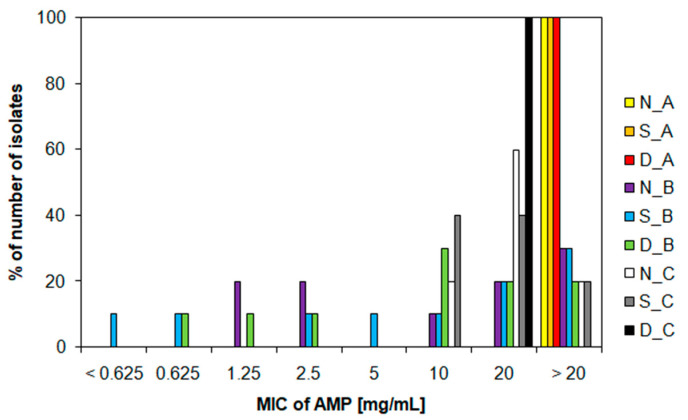
Frequency of minimum inhibitory concentration values for ampicillin determined in ampicillin-resistant isolates at different sampling campaign. AMP, ampicillin; MIC, minimum inhibitory concentration; N, nitrification tank; S, sedimentation tank; D, dam; A, December; B, August; C, November.

Ampicillin-resistant isolates were exposed to increasing concentrations of ampicillin and tetracycline and evaluated for MIC and MBC for these antibiotics. Ampicillin resistance patterns varied in different wastewater and dam water. Isolates from sampling campaigns A and C showed the highest resistance to ampicillin (*p* < 0.05) (Figure 4). All isolates from sampling campaign A showed MIC values >20 mg/mL (Figure 4). Moreover, 60% of the isolates from N_C and 40% of the isolates from S_C showed an MIC of 20 mg/mL (Figure 4). Other isolates showed an MIC of 10 mg/mL and below. Recent studies have shown different MIC values for bacterial isolates from wastewater: >1024 mg/L [50] and >256 μg/mL [51]. For a better overview of the diversity of persistent ampicillin-resistant bacteria, we determined the MBC values. They convinced us that the concentrations of ampicillin were not sufficient to completely kill the bacteria. One hundred percent of the isolates from sampling campaign A, from N_C and D_C, showed MBC values >20 mg/mL (Figure S2). Although the number of ARB was reduced during the WWTP cleaning process (Table 1) and this partially blocked the spread of ARB to the recipient, the results show that these surviving ARB carry a very high resistance to ampicillin (Figure 4) and there is also a large transfer of ARGs to the environment, especially the *bla*TEM gene (Figure 3).

**Figure 5:**
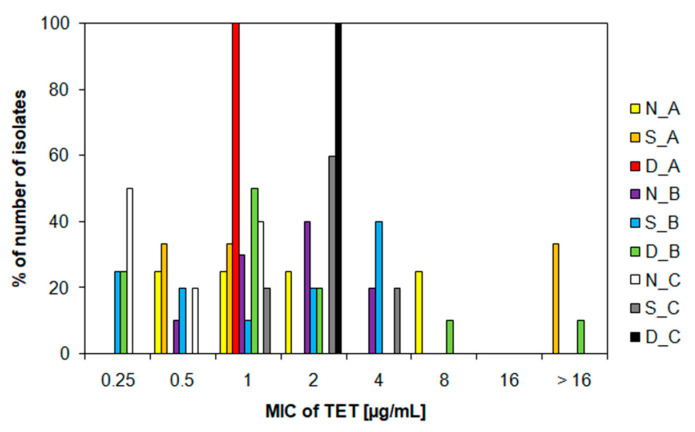
Frequency of minimum inhibitory concentration values for tetracycline determined in ampicillin-resistant isolates from the water samples at different sampling campaign. TET, tetracycline; MIC, minimum inhibitory concentration; N, nitrification tank; S, sedimentation tank; D, dam; A, December; B, August; C, November.

Furthermore, the MIC and MBC values for the antibiotic tetracycline were monitored in all isolates from water samples. MIC values for tetracycline were mostly in the range of 0.25–8.0 µg/mL, while for 33% of isolates from S_A and 10% of isolates from D_B, MIC values of >16 µg/mL were confirmed (Figure 5). These values agree with certain studies [51,52]. However, compared to other studies, our MIC values are somewhat lower. For example, Svobodová et al. (2018) discovered the presence of eight bacterial strains from Czech wastewater showing MICs for tetracycline >100 mg/mL [27]. Obayiuwana et al. (2018) found MIC values of 128 µg/mL in hospital wastewater isolates in Nigeria [50]. However, different MIC values for antibiotics may be related to other factors, such as the size of the WWTP or the wastewater source [53]. The analyzed MIC values did not show a sampling time relationship (*p* > 0.05) (Figure 5). MBC values were most often in the range of 0.5–8.0 µg/mL (Figure S3). Only 25% of isolates from N_A, 33% of isolates from S_A, and 10% of isolates from D_B had MBC values of >16 µg/mL (Figure S3). These results indicate that our studied WWTP probably accumulates ARB with lower resistance to the antibiotic tetracycline (Figure 5). Despite the higher level of tetracycline in the examined waters (Figure 2), there was no important increase or decrease in the abundance of the *tet*W gene (Figure 3).

### 3.4. Identification of Bacterial Isolates and Evaluation of Multidrug Resistance

All 49 bacterial isolates were identified by 16S rRNA sequencing. The sequence accession numbers from the NCBI database were shown in Table 2. Moreover, 89.8% of the isolates corresponded to the sequence *Aeromonas* sp. (Table 2); 6.1% of isolates (D4_B, D6_B, and D7_B) showed a similar sequence as *Pseudomonas* sp. Furthermore, 4.1% of isolates (S4_B, S10_B) were clustered as the sequence of *Morganella* sp. (Table 2). The occurrence of *Aeromonas* sp. in wastewater is not at all surprising. Due to their ubiquity in the aquatic environment, *Aeromonas* sp. is often used as a bacterial indicator of environmental antimicrobial resistance [54]. *Pseudomonas* sp. and *Morganella* sp. are well known as opportunistic hospital pathogens. This may contribute to their more frequent occurrence in the wastewater of the WWTP [55,56].

All identified bacterial isolates were tested for resistance genes that were already detected in water samples (see Section 3.2), i.e., *bla*TEM, *bla*NDM-1, *tet*W, and *van*A. The *bla*TEM gene was found in all isolates, which is clear evidence of its massive occurrence and spread in the environment (Table 2). The presence of the *bla*TEM gene was previously confirmed in *Aeromonas* sp., *Morganella* sp., and *Pseudomonas* sp. [57,58,59]. This is in line with our conclusion that large amounts of the *bla*TEM gene and beta-lactam-resistant bacteria accumulate in wastewater (Figure 3 and Figure 4). In contrast, the *bla*NDM-1 gene was found in nine isolates N1_A, N4_A, 2S_A, N8_B, S9_B, D6_B, D7_B, N2_C, and S3_C (Table 2). The *tet*W gene was also highly detected in isolates. It was not found in only eight isolates out of 49 (S3-A, N8_B, S3_B, D2_B, D8_B, D9_B, S2_C, and S4_C) (Table 2). The *van*A gene was found only in isolates D1_A, S2_C, and D1_C (Table 2). This may correlate with the fact that the *van*A gene was not detected at all in sampling campaign B (Figure 3).

Due to the detection of a larger number of genes in isolated bacterial strains from water samples, we were able to evaluate their multidrug resistance. Out of the total number of 49 isolated bacterial strains in 43 (i.e., 87.8%), two or more resistance genes were detected (Table 2). Such a high number is alarming; for example, Mukherjee et al. (2021), regarding the total number of isolated *E. coli*, found 19% of multidrug-resistant strains for two or more antibiotics [37]. As for our results, we found that most ampicillin-resistant isolates containing the *bla*TEM resistance gene have the potential for multidrug resistance with *tet*W (*p* < 0.05) (Table 2). However, other multidrug-resistant strains have been identified. Isolate S2_C contained the *bla*TEM and *van*A genes. In contrast, isolate N8_B contained *bla*TEM with *bla*NDM-1. Isolates D1_A and D1_C confirmed the presence of up to three resistance genes (*bla*TEM, *tet*W, and *van*A). The *bla*TEM, *bla*NDM-1, and *tet*W genes were detected simultaneously in strains N1_A, N4_A, S2_A, S9_B, D6_B, D7_B, N2_C, and S3_C (Table 2). The frequent occurrence of ARGs in wastewater isolates was also confirmed by Obayiuwana and Ibekwe (2020) [60]; however, the combination of resistance genes to beta-lactams, carbapenems, and tetracyclines at once is worrying (Table 2). It is very concerning that the two isolates identified as *Pseudomonas* sp. contained the *bla*TEM, *bla*NDM-1, and *tet*W genes (Table 2). *Pseudomonas* sp. is an opportunistic pathogen that causes severe urinary tract infections, gastrointestinal infections, diseases of the respiratory system, but is especially dangerous in patients with severe burns and weakened immunity [61,62]. The problem with this bacterial group is that it is able to colonize a large range of ecological environments, including wastewater and recreational water [63,64]. Once these multidrug-resistant *Pseudomonas* bacteria contaminate the food environment, they can reach humans and animals in the food chain in a variety of ways [65]. The multidrug resistance of opportunistic pathogens causes major problems in the treatment of infections and thus poses a significant threat to morbidity and mortality worldwide [61]. Confirmation of the presence of multidrug-resistant bacterial strains in wastewaters has only strengthened the hypothesis that horizontal gene transfer may occur between the same and different bacterial species [66]. We found that neither the sampling environment nor the different sampling times affected the development of multidrug resistance in isolated bacterial strains in wastewater and then dam water (*p* > 0.05). Other studies have detected significant development of multidrug-resistant bacteria during WWTP processes [67], but also beyond WWTPs [37]. It is known that a certain percentage of multidrug resistance exists naturally in bacteria [37]. By profiling multidrug resistance, in our study, we identified almost all isolates tested from wastewater and dam as being multidrug resistant (87.8%). These results clearly show the severity of the accumulation of antibiotics, ARB, and ARGs in the wastewater and during the WWTP treatment process. WWTPs thus significantly contribute to the development of multidrug-resistant bacteria and their spread to the environment. This finding highlights the need for more regular monitoring of the bacterial quality of wastewater, but also for further monitoring of the emergence and spread of last resort antibiotic resistance genes.

## 4. Conclusions

This study provided evidence of high ampicillin resistance in bacteria found in wastewater and also in purified water. Significant increases in the relative abundance of the *bla*TEM, *bla*NDM-1, and *van*A genes were found in the samples from the winter months of December and November. The *bla*TEM gene showed the highest relative abundance in dam water from all sampling sites and sampling campaigns. The number of ARB in the dam water decreased approximately three times contrary to the nitrification tank of the WWTP. An increased abundance of *bla*TEM means a massive occurrence of this gene in most bacteria in the environment. Ampicillin resistance was also supported by high MIC and MBC values in ampicillin-resistant isolates (61.2% of the total isolates had an MIC ≥ 20 mg/mL). In addition, multidrug-resistant strains were characterized. Remarkably, 87.8% of isolates contained two or more resistance genes. The *bla*TEM, *bla*NDM-1, and *tet*W genes were even detected in eight strains simultaneously. Strains that contained two or more resistance genes were identified as *Aeromonas* sp., *Morganella* sp., and *Pseudomonas* sp. These strains may contribute to the spread of multidrug resistance in the environment. However, there is not yet enough relevant information on the spread of multidrug resistance. Therefore, in the future, it will be necessary to develop and study the spread of multidrug resistance in the environment through cultivable and uncultivable microorganisms. Particular attention should be paid to last resort antibiotic resistance genes such as *bla*NDM-1 and *van*A genes. These genes pose a significant risk of failing antibiotic treatment.

## Figures and Tables

**Table 1:** Calculation of CFU/mL in the water samples at different sampling campaigns. The bacteria were loaded on the pure TSA medium and TSA containing 500 mg/L of ampicillin. Plated samples were incubated at 30 °C for 24 h. AMP, ampicillin; WWTP, wastewater treatment plant; A, December; B, August; C, November. Capital letters indicate the significant difference between the sampling times (*p* < 0.05). Lowercase letters indicate the significant differences between the different sampling sites (*p* < 0.05). * indicates the significant difference between the resistant and non-resistant bacteria (*p* < 0.05).

Sampling	Water Sample	Without AMP	500 mg/L AMP
		log CFU/mL	
A	Nitrification tank WWTP	6.410 ± 0.067 ^Abc^	6.006 ± 0.015 ^Abc^
	Sedimentation tank WWTP	3.452 ± 0.054 ^a^	2.694 ± 0.013 * ^a^
	Dam	3.034 ± 0.080 ^a^	2.041 ± 0.001 * ^a^
B	Nitrification tank WWTP	6.300 ± 0.022 ^bc^	5.581 ± 0.062 * ^bc^
	Sedimentation tank WWTP	4.019 ± 0.023 ^a^	3.423 ± 0.025 * ^a^
	Dam	3.349 ± 0.048 ^a^	2.292 ± 0.088 * ^a^
C	Nitrification tank WWTP	6.067 ± 0.164 ^bc^	5.818 ± 0.195 ^bc^
	Sedimentation tank WWTP	3.554 ± 0.239 ^a^	2.970 ± 0.099 ^a^
	Dam	2.540 ± 0.062 ^a^	1.739 ± 0.040 * ^a^

**Table 2:** Sequence identification of ampicillin-resistant isolates and detection of antibiotic resistance genes (*bla*TEM, *bla*NDM-1, *tet*W, and *van*A) in the ampicillin-resistant isolates from the water samples at different sampling campaigns. Accession numbers of individual isolates come from the NCBI GeneBank database. N, nitrification tank; S, sedimentation tank; D, dam; A, December; B, August; C, November.

Isolate	*bla*TEM	*bla*NDM-1	*tet*W	*van*A	Identification	Accession Number
N1_A	+	+	+	-	*Aeromonas* sp.	OL832061
N2_A	+	-	+	-	*Aeromonas* sp.	OL832062
N3_A	+	-	+	-	*Aeromonas* sp.	OL832063
N4_A	+	+	+	-	*Aeromonas* sp.	OL832064
S1_A	+	-	+	-	*Aeromonas* sp.	OL832065
S2_A	+	+	+	-	*Aeromonas* sp.	OL832066
S3_A	+	-	-	-	*Aeromonas* sp.	OL832067
D1_A	+	-	+	+	*Aeromonas* sp.	OL832068
N1_B	+	-	+	-	*Aeromonas* sp.	OL832069
N2_B	+	-	+	-	*Aeromonas* sp.	OL832070
N3_B	+	-	+	-	*Aeromonas* sp.	OL832071
N4_B	+	-	+	-	*Aeromonas* sp.	OL832072
N5_B	+	-	+	-	*Aeromonas* sp.	OL832073
N6_B	+	-	+	-	*Aeromonas* sp.	OL832074
N7_B	+	-	+	-	*Aeromonas* sp.	OL832075
N8_B	+	+	-	-	*Aeromonas* sp.	OL832076
N9_B	+	-	+	-	*Aeromonas* sp.	OL832077
N10_B	+	-	+	-	*Aeromonas* sp.	OL832078
S1_B	+	-	+	-	*Aeromonas* sp.	OL832079
S2_B	+	-	+	-	*Aeromonas* sp.	OL832080
S3_B	+	-	-	-	*Aeromonas* sp.	OL832081
S4_B	+	-	+	-	*Morganella* sp.	OL832082
S5_B	+	-	+	-	*Aeromonas* sp.	OL832083
S6_B	+	-	+	-	*Aeromonas* sp.	OL832084
S7_B	+	-	+	-	*Aeromonas* sp.	OL832085
S8_B	+	-	+	-	*Aeromonas* sp.	OL832086
S9_B	+	+	+	-	*Aeromonas* sp.	OL832087
S10_B	+	-	+	-	*Morganella* sp.	OL832088
D1_B	+	-	+	-	*Aeromonas* sp.	OL832089
D2_B	+	-	-	-	*Aeromonas* sp.	OL832090
D3_B	+	-	+	-	*Aeromonas* sp.	OL832091
D4_B	+	-	+	-	*Pseudomonas* sp.	OL832092
D5_B	+	-	+	-	*Aeromonas* sp.	OL832093
D6_B	+	+	+	-	*Pseudomonas* sp.	OL832094
D7_B	+	+	+	-	*Pseudomonas* sp.	OL832095
D8_B	+	-	-	-	*Aeromonas* sp.	OL832096
D9_B	+	-	-	-	*Aeromonas* sp.	OL832097
D10_B	+	-	+	-	*Aeromonas* sp.	OL832098
N1_C	+	-	+	-	*Aeromonas* sp.	OL832099
N2_C	+	+	+	-	*Aeromonas* sp.	OL832100
N3_C	+	-	+	-	*Aeromonas* sp.	OL832101
N4_C	+	-	+	-	*Aeromonas* sp.	OL832102
N5_C	+	-	+	-	*Aeromonas* sp.	OL832103
S1_C	+	-	+	-	*Aeromonas* sp.	OL832104
S2_C	+	-	-	+	*Aeromonas* sp.	OL832105
S3_C	+	+	+	-	*Aeromonas* sp.	OL832106
S4_C	+	-	-	-	*Aeromonas* sp.	OL832107
S5_C	+	-	+	-	*Aeromonas* sp.	OL832108
D1_C	+	-	+	+	*Aeromonas* sp.	OL832109

## Data Availability

The sequences used in this study have been deposited in the NCBI GeneBank database.

## Supplementary Materials

The following are available online at https://www.mdpi.com/article/10.3390/life12020147/s1, Table S1: Sequences of primers and PCR conditions used in the study, Table S2: Efficiency of qPCR assays retrieved from standard curves. Sampling times: A—December, B—August, C—November, Table S3: Growth curves parameters of ampicillin-resistant isolates from the nitrification and sedimentation tanks of the wastewater treatment plant and dam at different sampling campaign (A—December, B—August, C—November). The growth curves measured every 30 min 24 h at 600 nm and 30 °C. N, nitrification tank; S, sedimentation tank; D, dam, Figure S1: Growth curves of ampicillin-resistant isolates from the nitrification and sedimentation tanks of the wastewater treatment plant and dam at different sampling campaign (A—December, B—August, C—November). The growth curves measured every 30 min 24 h at 600 nm and 30 °C. N, nitrification tank; S, sedimentation tank; D, dam, Figure S2: Frequency of minimum bactericidal concentration values for ampicillin determined in ampicillin-resistant isolates from the water samples from the nitrification and sedimentation tanks of the wastewater treatment plant and dam at different sampling campaign (A—December, B—August, C—November). AMP, ampicillin; MBC, minimum bactericidal concentration; N, nitrification tank; S, sedimentation tank; D, dam, Figure S3: Frequency of minimum bactericidal concentration values for tetracycline determined in ampicillin-resistant isolates from the water samples from the nitrification and sedimentation tanks of the wastewater treatment plant and dam at different sampling campaign (A—December, B—August, C—November). TET, tetracycline; MBC, minimum bactericidal concentration; N, nitrification tank; S, sedimentation tank; D, dam.
