# Supplementary material for: Resistant Genes and Multidrug-Resistant Bacteria in Wastewater: A Study of Their Transfer to the Water Reservoir in the Czech Republic
Source: Life (Basel). 2022 Jan 20;12(2):147. doi: 10.3390/life12020147 (PMC8875776; doi:10.3390/life12020147)
Supplement: Supplementary file 1 [file life-12-00147-s001.zip › life-1540837-supplementary.pdf]

Article

Resistant Genes and Multidrug-Resistant Bacteria in Wastewater: A Study of Their Transfer to the Water Reservoir in the Czech Republic

Tereza Stachurová , Nikola Sýkorová, Jaroslav Semerád and Kateřina Malachová

Supplementary Materials:

Table S1. Sequences of primers and PCR conditions used in the study.

Gene	Primer	5‘-3‘ Sequence	Standard PCR Conditions	Reference
16S rRNA	F1048	GTGSTGCAYGGYTGTCGTCA	94°C 3 min, 40× (94°C 15 s,60°C 60 s)	Marti et al. 2013
	R1194	ACGTCRTCCM-CACCTTCCTC		
16S rRNA (identification of bacteria)	984F	AACGCGAAGAAC-CTTAC	95°C 2 min, 35× (95°C 45s, 55°C 30 s, 72°C 60 s), 72°C 10 min	Heuer et al. 1997
	1378R	CGGTGTG-TACAAGGCCCGG-GAACG		
*bla*TEM	bla-TEM-RX	CTTTATCCGCCTCCATCCAGTCTA	94°C 2 min, 40× (94°C 15s,60°C 30s, 72°C 45 s), 72°C 10 min	Marti et al. 2013
	bla-TEM-FX	GCK-GCCAACTTACTTCTGACAACG		
*bla*NDM-1	NDM-Rm	CGGAATGGCTCATCACGATC	94°C 2 min, 40× (94°C 15s,53°C 30 s, 72°C 45 s), 72°C 10 min	Poirel et al. 2011
	NDM-Fm	GGTTT-GGCGATCTGGTTTTC		
*bla*OXA-48	bla-OXA-48-R	GAGCACTTCTTTT-GTGATGGC	95°C 5 min, 35× (95°C 1 min, 56°C 1 min, 72°C 1 min), 72°C 5 min	Mlynarcik et al. 2016
	bla-OXA-48-F	TTGGTGG-CATCGATTATCGG		
*bla*KPC	bla-KPC-R	TTACTGCCCGTTGA CGCCC	94°C 3 min, 30× (94°C 1 min, 55°C 1 min, 72°C 1 min), 72°C 5 min	Ribeiro et al. 2016
	bla-KPC-F	ATGTCACTG-TATCGCCGTCT		
*mec*A	mecA-LP	GATAGCAGTTA-TATTTCTA	95°C 5 min, 35× (94°C 15 s,48°C 60 s, 72°C 80 s), 72°C 4 min	Colomer-Lluch et al. 2011
	mecA-UP	ATACTTAG-TTCTTTAGCGAT		
*tet*W	tet(W)-RV	GGGCG-TATCCACAATGTTAAC	94°C 2 min, 40× (94°C 15 s,60°C 30 s, 72°C 45 s), 72°C 10 min	Marti et al. 2013
	tet(W)-FW	GAGAGCCTGCTA-TATGCCAGC		
*van*A	vanA-R	GATTCCGTACTG-CAGCCTGATT	94°C 3min, 40× (94°C 15 s,60°C 30 s, 72°C 60 s), 72°C 10 min	Rathnayake et al. 2012
	vanA-F	TGTGCGG-TATTGGGAAACAG		

Table S2. Efficiency of qPCR assays retrieved from standard curves. Sampling times: A – December, B – August, C – November.

Sampling	qPCR Assay	Efficiency [%]	R^2	Limit of Quantification (Copy Number)
	rDNA	97.64	0.996	20.1
	*bla*TEM	96.24	0.995	22.5
A	*bla*NDM-1	99.26	0.997	27.9
	*tet*W	98.75	0.993	24.4
	*van*A	95.99	0.995	27.5
	rDNA	95.98	0.995	20.4
	*bla*TEM	96.73	0.996	22.1
B	*bla*NDM-1	97.11	0.994	27.8
	*tet*W	96.08	0.997	23.8
	*van*A	97.42	0.994	28.3
	rDNA	96.18	0.997	19.9
	*bla*TEM	97.16	0.998	21.8
C	*bla*NDM-1	98.75	0.997	28.2
	*tet*W	92.66	0.996	24.1
	*van*A	96.09	0.997	27.6

Table S3. Growth curves parameters of ampicillin-resistant isolates from the nitrification and sedimentation tanks of the wastewater treatment plant and dam at different sampling campaign (A – December, B – August, C – November). The growth curves measured every 30 min 24 h at 600 nm and 30 °C. N, nitrification tank; S, sedimentation tank; D, dam.

Isolate	Growth Rate (h)	lag Phase (h)	Doubling Time (h)
N1_A	1.388 ± 0.006	2.146 ± 0.116	0.500 ± 0.002
N2_A	0.794 ± 0.157	10.893 ± 0.094	0.897 ± 0.087
N3_A	1.325 ± 0.064	5.896 ± 0.178	0.524 ± 0.025
N4_A	0.649 ± 0.053	9.152 ± 0.085	1.072 ± 0.091
S1_A	0.403 ± 0.005	5.382 ± 0.272	1.718 ± 0.023
S2_A	0.804 ± 0.147	2.897 ± 0.149	0.880 ± 0.152
S3_A	0.589 ± 0.136	7.288 ± 0.167	1.217 ± 0.261
D1_A	0.714 ± 0.010	11.239 ± 0.086	0.971 ± 0.013
N1_B	4.242 ± 0.063	12.073 ± 0.140	0.160 ± 0.002
N2_B	6.584 ± 0.065	0.556 ± 0.087	0.100 ± 0.001
N3_B	0.646 ± 0.062	8.886 ± 0.113	1.079 ± 0.099
N4_B	7.739 ± 0.280	1.982 ± 0.212	0.089 ± 0.005
N5_B	1.227 ± 0.052	10.407 ± 0.062	0.560 ± 0.002
N6_B	1.113 ± 0.083	9.927 ± 0.132	0.625 ± 0.045
N7_B	5.873 ± 0.102	0.747 ± 0.018	0.110 ± 0.002
N8_B	0.508 ± 0.129	5.427 ± 0.098	1.422 ± 0.350
N9_B	1.396 ± 0.023	4.956 ± 0.079	0.490 ± 0.008
N10_B	0.438 ± 0.016	6.182 ± 0.061	1.584 ± 0.057
S1_B	6.191 ± 0.060	0.779 ± 0.065	0.110 ± 0.001
S2_B	5.515 ± 0.780	1.859 ± 0.143	0.130 ± 0.007
S3_B	3.194 ± 0.321	8.442 ± 0.329	0.210 ± 0.002
S4_B	7.067 ± 0.379	0.968 ± 0.099	0.100 ± 0.005
S5_B	5.621 ± 0.102	0.813 ± 0.079	0.120 ± 0.002
S6_B	5.006 ± 0.027	0.951 ± 0.024	0.140 ± 0.007
S7_B	3.068 ± 0.241	0.853 ± 0.035	0.227 ± 0.018
S8_B	0.792 ± 0.173	7.465 ± 0.200	0.901 ± 0.180
S9_B	5.031 ± 0.170	1.522 ± 0.364	0.140 ± 0.004
S10_B	7.308 ± 0.540	1.636 ± 0.252	0.095 ± 0.002
D1_B	2.998 ± 0.131	4.466 ± 0.271	0.230 ± 0.009
D2_B	5.980 ± 0.553	0.799 ± 0.013	0.120 ± 0.001
D3_B	4.614 ± 0.044	1.239 ± 0.133	0.150 ± 0.006
D4_B	3.107 ± 0.063	5.829 ± 0.155	0.220 ± 0.004
D5_B	5.648 ± 0.145	2.154 ± 0.346	0.120 ± 0.002
D6_B	3.211 ± 0.042	5.844 ± 0.156	0.220 ± 0.005
D7_B	2.903 ± 0.019	5.855 ± 0.182	0.240 ± 0.001

D8_B	9.808 ± 0.574	0.635 ± 0.049	0.071 ± 0.001
D9_B	5.648 ± 0.078	1.228 ± 0.126	0.120 ± 0.002
D10_B	5.349 ± 0.551	1.651 ± 0.294	0.130 ± 0.001
N1_C	1.619 ± 0.002	0.100 ± 0.001	42.810 ± 0.309
N2_C	4.997 ± 0.006	4.941 ± 0.209	13.873 ± 0.163
N3_C	1.932 ± 0.006	0.201 ± 0.043	35.891 ± 0.110
N4_C	2.421 ± 0.004	1.078 ± 0.105	28.632 ± 0.452
N5_C	1.408 ± 0.001	0.386 ± 0.042	49.233 ± 0.171
S1_C	1.505 ± 0.018	8.428 ± 0.151	46.524 ± 0.578
S2_C	0.969 ± 0.002	13.848 ± 0.319	74.281 ± 0.674
S3_C	1.964 ± 0.001	0.010 ± 0.001	35.285 ± 0.256
S4_C	1.812 ± 0.003	4.319 ± 0.249	38.251 ± 0.731
S5_C	2.948 ± 0.015	0.015 ± 0.002	23.556 ± 0.126
D1_C	1.136 ± 0.002	10.282 ± 0.161	61.001 ± 1.113

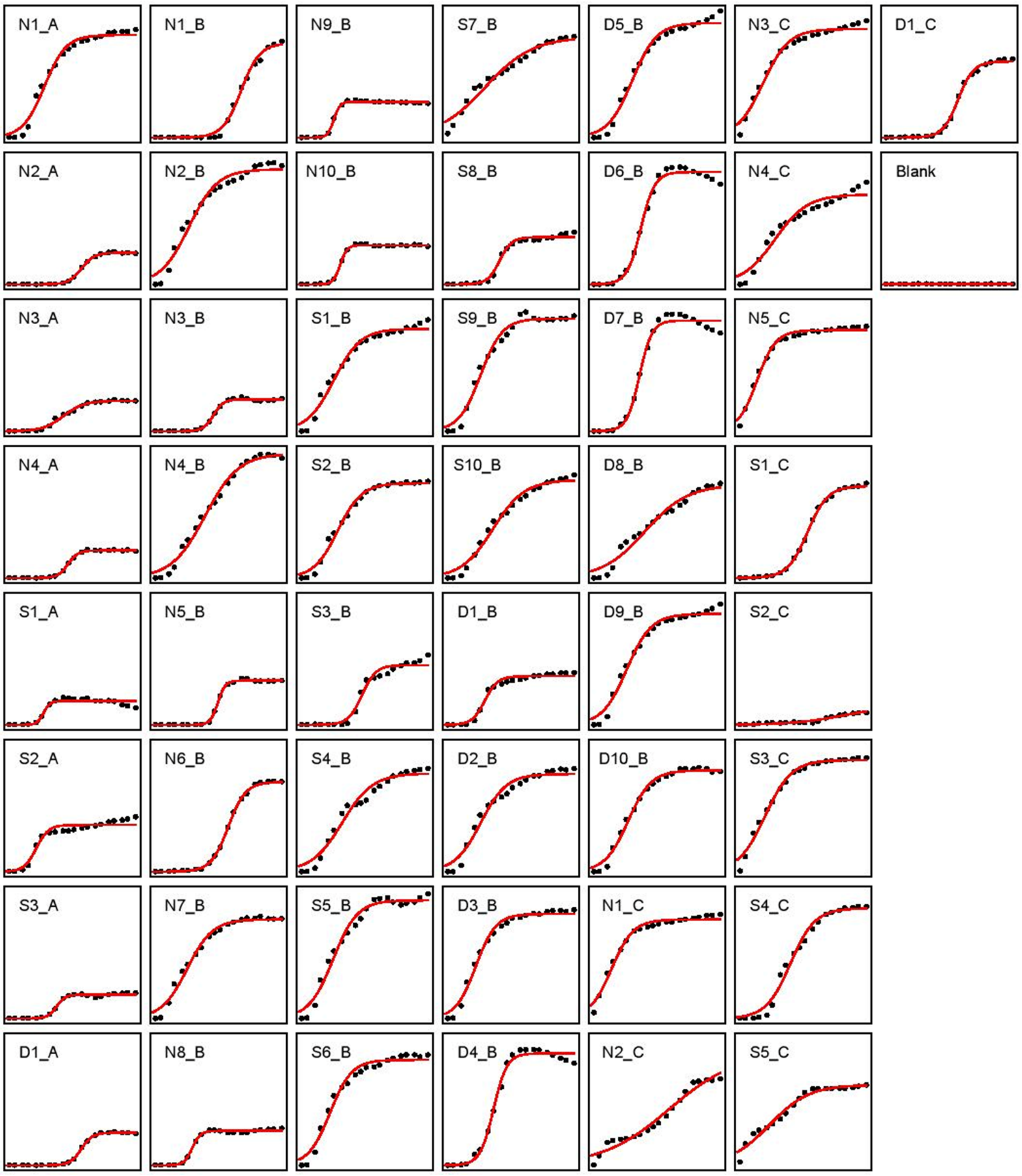

Figure S1. Growth curves of ampicillin-resistant isolates from the nitrification and sedimentation tanks of the wastewater treatment plant and dam at different sampling campaign (A – December, B – August, C – November). The growth curves measured every 30 min 24 h at 600 nm and 30 °C. N, nitrification tank; S, sedimentation tank; D, dam.

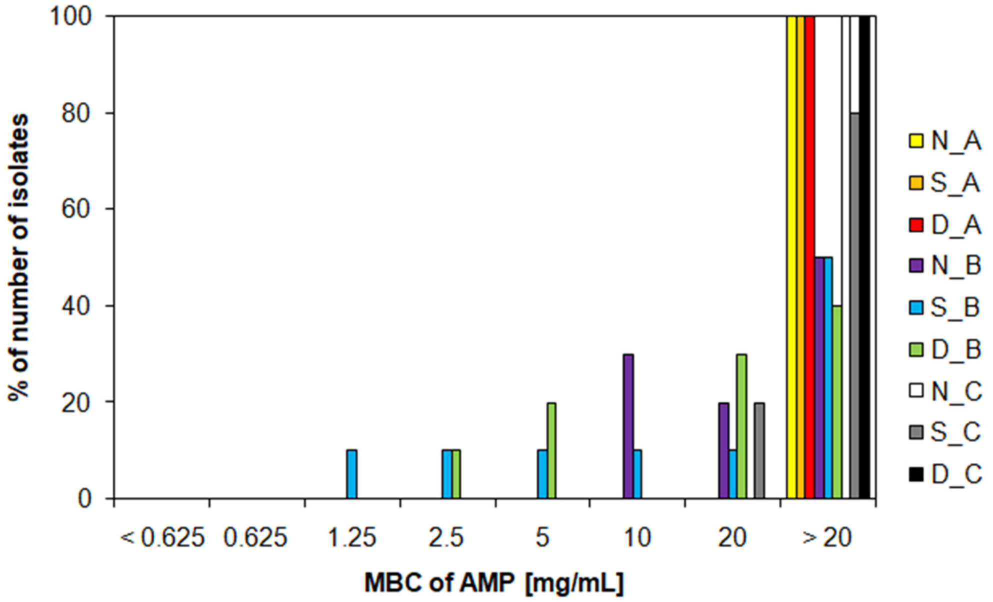

Figure S2. Frequency of minimum bactericidal concentration values for ampicillin determined in ampicillin-resistant isolates from the water samples from the nitrification and sedimentation tanks of the wastewater treatment plant and dam at different sampling campaign (A – December, B – August, C – November). AMP, ampicillin; MBC, minimum bactericidal concentration; N, nitrification tank; S, sedimentation tank; D, dam.

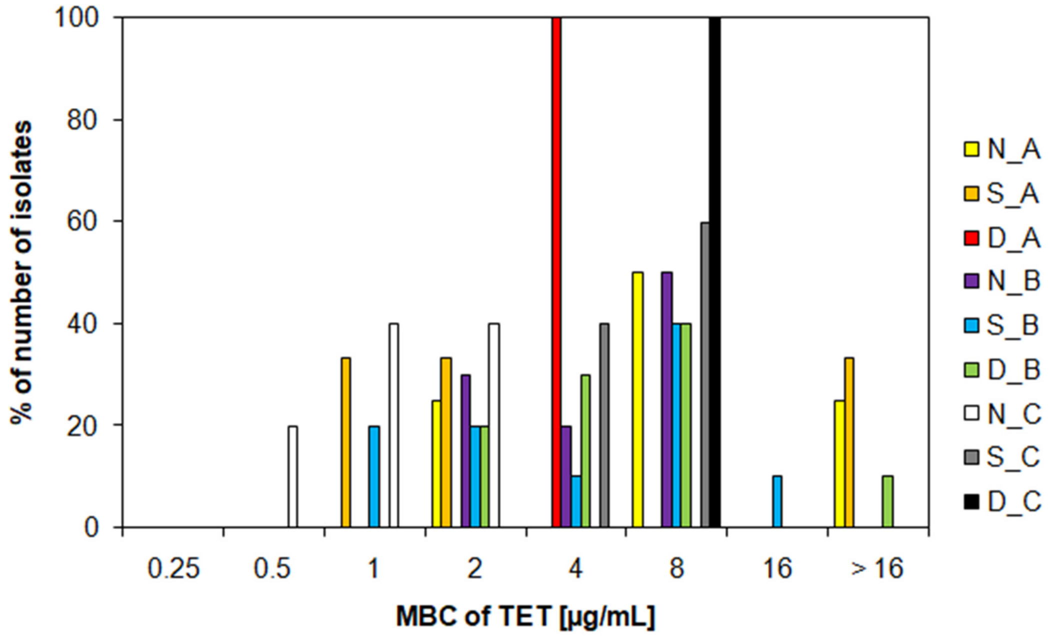

Figure S3. Frequency of minimum bactericidal concentration values for tetracycline determined in ampicillin-resistant isolates from the water samples from the nitrification and sedimentation tanks of the wastewater treatment plant and dam at different sampling campaign (A – December, B – August, C – November). TET, tetracycline; MBC, minimum bactericidal concentration; N, nitrification tank; S, sedimentation tank; D, dam.
